# π-Electron-Extended Triazine-Based Covalent Organic Framework as Photocatalyst for Organic Pollution Degradation and H_2_ Production from Water

**DOI:** 10.3390/polym15071685

**Published:** 2023-03-28

**Authors:** Jing Han Wang, Taher A. Gaber, Shiao-Wei Kuo, Ahmed F. M. EL-Mahdy

**Affiliations:** 1 Department of Materials and Optoelectronic Science, National Sun Yat-sen University, Kaohsiung 80424, Taiwan; m103100001@mail.nsysu.edu.tw (J.H.W.); d113100008@mail.nsysu.edu.tw (T.A.G.); kuosw@faculty.nsysu.edu.tw (S.-W.K.); 2 Chemistry Department, Faculty of Science, Assiut University, Assiut 71516, Egypt

**Keywords:** covalent organic framework, rhodamine B, dye removal, photodegradation, photocatalysis H_2_ evolution

## Abstract

Herein, we report the efficient preparation of π-electron-extended triazine-based covalent organic framework (TFP-TPTPh COF) for photocatalysis and adsorption of the rhodamine B (RhB) dye molecule, as well as for photocatalytic hydrogen generation from water. The resultant TFP-TPTPh COF exhibited remarkable porosity, excellent crystallinity, high surface area of 724 m^2^ g^−1^, and massive thermal stability with a char yield of 63.41%. The TFP-TPTPh COF demonstrated an excellent removal efficiency of RhB from water in 60 min when used as an adsorbent, and its maximum adsorption capacity (*Q*_m_) of 480 mg g^−1^ is among the highest *Q*_m_ values for porous polymers ever to be recorded. In addition, the TFP-TPTPh COF showed a remarkable photocatalytic degradation of RhB dye molecules with a reaction rate constant of 4.1 × 10^−2^ min^−1^ and an efficiency of 97.02% under ultraviolet–visible light irradiation. Furthermore, without additional co-catalysts, the TFP-TPTPh COF displayed an excellent photocatalytic capacity for reducing water to generate H_2_ with a hydrogen evolution rate (HER) of 2712 μmol g^−1^ h^−1^. This highly active COF-based photocatalyst appears to be a useful material for dye removal from water, as well as solar energy processing and conversion.

## 1. Introduction

The scarcity of clean water sources has long existed because of industrialization, rapid development, and anthropogenic activity [1]. The development of feasible systems for preventing water pollution and protecting the environment has attracted the attention of researchers [2]. Among the most prevalent water pollutants are organic dyes. Due to their chemical structures, dyes are resistant to light, water, and many synthetic substances, making them non-biodegradable [3]. In addition, organic dyes are mutagenic and carcinogenic substances that can potentially seriously harm human health and the environment [4]. Researchers recently invented several wastewater treatment technologies for eliminating organic dyes from wastewater, including chemical, physical, and biological technologies [5,6,7,8]. Among them, sorption-based technologies received more attention due to their cost-effectiveness [9,10]. Amorphous porous materials, such as natural zeolites, fibers, and nanoscale-ordered porous materials, have been invented as dye adsorbents [11,12,13,14,15,16,17,18]. These porous sorbents, however, only adsorb contaminated dyes without degrading them, causing regeneration and secondary pollution issues [19]. However, recent research has focused on porous photocatalytic materials as potent new technological advances for removing and degrading polluted organic dyes from water [20,21,22,23,24]. Many inorganic porous materials, such as metal sulfides and oxides, have been investigated as photocatalysts for organic dye photodegradation. Still, their minimal surface areas and rising toxic effects have severely restricted their practicality [25,26,27,28,29,30]. As a direct consequence, that is crucial to create innovative sorbents that can effectively adsorb and photodegrade polluting dyes under visible light.

In the last ten years, porous organic polymers have become an exciting subject of photocatalysts, due to their benefits in molecular and controllable electronic structures [31,32,33]. The reported porous polymer photocatalysts include covalent triazine frameworks, conjugated microporous polymers, and porous aromatic frameworks [34,35,36,37,38]. In fact, researchers developed several porous organic polymers with various topologies and morphological characteristics for photodegrading organic polluted dyes [39,40,41]. Among these porous organic polymers, covalent organic frameworks (COFs) have benefits of elevated crystallinity, high surface area, low density, precisely controllable structural features, facilely tailored functionality, tunable pore size and structure, and a gifted covalent combination of building units, and they can absorb and move light energy via their own delocalized polymer backbones [41,42,43,44,45]. The majority of these COFs demonstrated significant characteristics. Then, they excelled in a variety of uses in gas uptaking, energy storage, poisonous metal ion sensing and removing, detecting, photocatalysis, optical devices, and solar energy [46,47,48,49,50,51,52,53,54]. Additionally, COFs are desirable substances for the adsorptive and photocatalytic degradation of natural dyes in aqueous media due to their significant absorption activities and extensive conjugation [55,56,57]. Only a few reports have mentioned using COFs as photocatalysts to degrade organic pollutants, even though COFs have been extensively studied as photocatalysts for organic reactions and hydrogen production via water splitting [58,59]. Therefore, the creation of environmentally friendly COFs as semiconductor materials for the adsorptive and photocatalytic degradation of natural dyes in aqueous media is still a challenge.

**Scheme 1:**
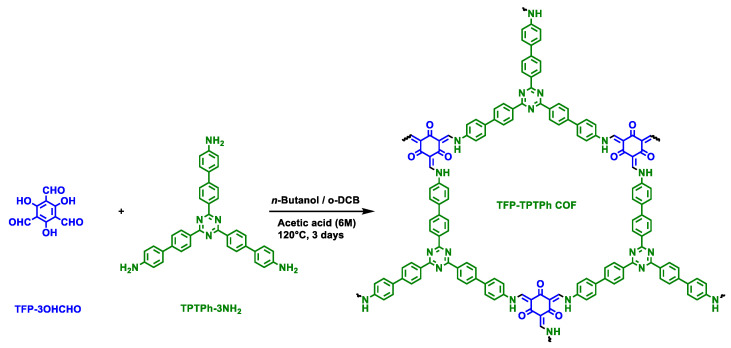
Synthesis of TFP-TPTPh COF.

It is believed that a 1,3,5-triazine unit correlated significantly with high photocatalytic efficacy in organic photocatalysts [60]. According to reports, extending the π-electron delocalization system of COFs significantly improved their electronic and optical attributes [15]. Along these lines, we developed π-electron extended triazine-based COF (TFP-TPTPh COF) through Schiff-base polycondensation of 1,3,5-triformylphloroglucinol (TFP-3OHCHO) with π-electron-extended 4′,4‴,4′′′′′-(1,3,5-triazine-2,4,6-triyl)tris(([1,1′-biphenyl]-4-amine)) (TPTPh-3NH_2_) under solvothermal condition (Scheme 1). After that, the resultant triazine-based COF was evaluated as a promising adsorbent and photocatalyst for the removal and photocatalytic degradation of RhB dye molecules in water. In addition, the obtained triazine-based COF was a photocatalyst for the photocatalytic reduction of water to yield H_2_.

## 2. Materials and Methods

### 2.1. Materials

Chemicals were acquired from commercial sources and used as obtained. Phloroglucinol (99%), potassium carbonate (K_2_CO_3_), nitrobenzene, 4-aminophenylboronic acid pinacol ester, acetic acid (CH_3_COOH), and *n*-butanol were ordered from Sigma–Aldrich (St. Louis, MO, USA). Tetrakis(triphenylphosphine)palladium(0) (Pd(PPh_3_)_4_), trifluoromethanesulfonic acid (≥98%), *o*-dichlorobenzene, and bromobenzonitrile (≥98%) were obtained from Acros (Maribor, Slovenia).

### 2.2. Synthesis of 4′,4‴,4′′′′′-(1,3,5-Triazine-2,4,6-triyl)tris(([1,1′-biphenyl]-4-amine)) (TPTPh-3NH_2_)

In a 250 mL glass bottle and under an inert atmosphere, 2,4,6-tris(4-bromopheny)-1,3,5-triazine (TPT-3Br) (0.4 g, 0.73 mmol, one equivalent), 4-aminophenylboronic acid pinacol ester (0.97 g, 4.43 mmol, six equivalent), Pd(PPh_3_)_4_ (86 mg, 0.073 mmol, 0.1 equivalent), and K_2_CO_3_ (0.76 g, 5.5 mmol, 7.5 equivalent) were weighted. Then, dioxane/water (30 mL: 7.5 mL) was added, and the reaction was allowed to heat at 115 °C for three days. After pouring into ice water (50 mL), the TPTPh-3NH_2_ was separated by filtration to obtain it as a green-brown powder (0.34 g, 80%).

### 2.3. Synthesis of TFP-TPTPh COF

TFP-3OHCHO (40 mg, 0.19 mmol) and TPTPh-3NH_2_ (111 mg, 0.19 mmol) were weighed into a Schlenk tube containing a co-solvent of *n*-butanol (5 mL)/o-dichlorobenzene (5 mL) and AcOH (6 M, 1 mL). The reaction mixture was flame-sealed and allowed to heat at 120 °C for three days. The solid TFP-TPTPh COF was filtered, washed twice with *n*-butanol, and dried under vacuum at 120 °C to acquire TFP-TPTPh COF as a yellow powder.

## 3. Results

### 3.1. Design, Synthesis, and Crystallinity of π-Electron-Extended Triazine-Based COF

We synthesized the π-electron-extended TPTPh-3NH_2_ building monomer through a simple Suzuki-coupling reaction of TPT-3Br with 4-aminophenylboronic acid pinacol ester in the presence of Pd(PPh_3_)_4_ as a Pd-catalyst and K_2_CO_3_ as a basic catalyst at 115 °C using a co-solvent of 1,4-dioxane and water (Scheme S1). Fourier-transform infrared (FTIR) and nuclear magnetic resonance (NMR) spectroscopies were performed to figure out the molecular structure of the TPTPh-3NH_2_ monomer (Figures S1–S3). The FTIR spectrum of TPT-3PhNH_2_ displayed the N–H bond from 3446 to 3326 cm^−1^, the aromatic C–H stretching at 3071 cm^−1^, the C=N bond at 1600 cm^−1^, the aromatic C=C bond at 1509 cm^−1^, and the C–N bond at 1281 cm^−1^ (Figure S1). As shown in Figure S2, the characteristic ^1^H signals of the aromatic ring were observed at 7.85 ppm (a), 7.56 ppm (b), and 6.69 ppm (c). The signal appeared at 5.45 ppm for the NH_2_ group. The ^13^C NMR spectrum of TPT-3PhNH_2_ showed a signal at 171.46 ppm (a) that could be assigned due to the carbon of the triazine functional group and a signal at 150.21 ppm (b) for the carbon nuclei attached to amino groups (C–NH_2_). The peaks located around 145.71 ppm to 114.79 ppm (c-i) are derived from the carbon atoms of the aromatic rings (Figure S3). Scheme 1 reveals the solvothermal synthesis and construction of the π-electron extended triazine-based COF (TFP-TPTPh COF) from the TPTPh-3NH_2_ and TFP-3OHCHO (Scheme S2, Figures S4–S6) building monomers. Briefly, the polycondensation between TPTPh-3NH_2_ and TFP-3OHCHO in a mixed solvent of o-dichlorobenzene, *n*-butanol, and acetic acid (6 M) (1:1:0.1) at 120 °C for three days afforded the desired TFP-TPTPh COF in high yield.

**Figure 1:**
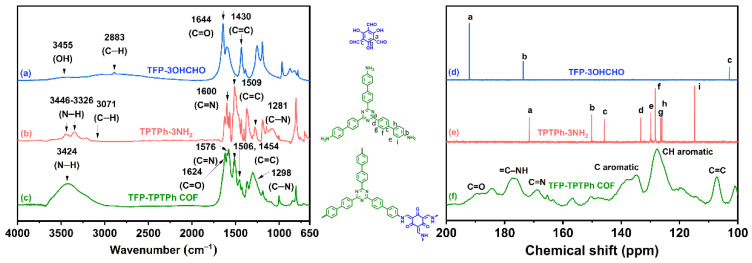
(**a**–**c**) FTIR spectra of (**a**) TFP-3OHCHO, (**b**) TPTPh-3NH_2_, and (**c**) TFP-TPTPh COF. (**d**–**f**) ^13^C SS-NMR spectra of (**d**) TFP-3OHCHO, (**e**) TPTPh-3NH_2_, and (**f**) TFP-TPTPh COF.

FTIR and solid-state ^13^C NMR (^13^C SS-NMR) spectroscopies were used to verify the molecular composition of the as-prepared TFP-TPTPh COF. The FTIR spectrum of TFP-TPT COF was expressed in Figure 1a–c, and there were spectra of the monomer comparison to exhibit the growth and decline of the peak. TFP-3OHCHO displayed two intense signals for CH=O and C=C units at 1644 and 1430 cm^−1^, respectively, as well as a robust and broad absorption at 3455 cm^−1^ for the hydroxyl function groups (Figure 1a). The FTIR spectrum of the TPTPh-3NH_2_ characterized signals at 3446–3326 cm^−1^ for the amine function units, at 1600 cm^−1^ for imine (C=N) groups, at 1509 cm^−1^ for aromatic (C=C) stretching, and at 1281 cm^−1^ for the C–N units (Figure 1b). The FTIR spectrum of the TFP-TPTPh COF was short of any signal for the OH groups of the TFP-3OHCHO or the NH_2_ groups of TPTPh-3NH_2_, indicating that they had been completely consumed. The presence of a robust peak at 3424 cm^−1^ for the N–H function units, along with peaks at 1624 cm^−1^ for the C=O function units, 1576 cm^−1^ for the imine C=N function units, 1506~1454 cm^−1^ for the covalently C=C units, and 1298 cm^−1^ for the C–N function units, verified that TFP-TPTPh COF existed in β-keto-enamine form (Figure 1c). The ^13^C SS-NMR spectrum of TFP- TPTPh COF is displayed in Figure 1d–f. For compression, the ^13^C NMR spectrum of TFP-3OHCHO exhibited a signal at 192.05 ppm for the aldehydic (CH=O) carbons and a signal at 173.59 ppm for the phenolic (C–OH) carbons (Figure 1d). The ^13^C NMR spectrum of TPT-3PhNH_2_ showed a signal at 150.21 ppm for the carbon nuclei attached to amino groups (C–NH_2_) (Figure 1e). The signal of C–OH carbon atom disappeared after the polycondensation with the TPT-3PhNH_2_. In addition, the ^13^C SS-NMR spectrum of TFP- TPTPh COF showed signals at 189.14–186.68 ppm for the keto (C=O) units. The enamine (=C–NH) and α-enamine (C=C) carbon atoms appeared in the range between 177.01–175.73 ppm and 107.48–107.16 ppm, confirming the consistency of the β-keto-enamine-linked COF (Figure 1f). The exceptional thermal stability of the TFP-TPTPh COF proved its high degree of polycondensation. Figure S7 and Table S1 show that TFP-TPTPh COF is highly thermally stable, as evaluated by thermogravimetric analysis at temperatures ranging from 40 to 800 °C in a nitrogen atmosphere. The as-synthesized TFP-TPTPh COF retained approximately 90% of its original mass after heating to 414.33 °C; after heating to 800 °C, the char yield was 63.41%.

### 3.2. BET, XRD, FE-SEM, and TEM of π-Electron-Extended Triazine-Based COF

**Figure 2:**
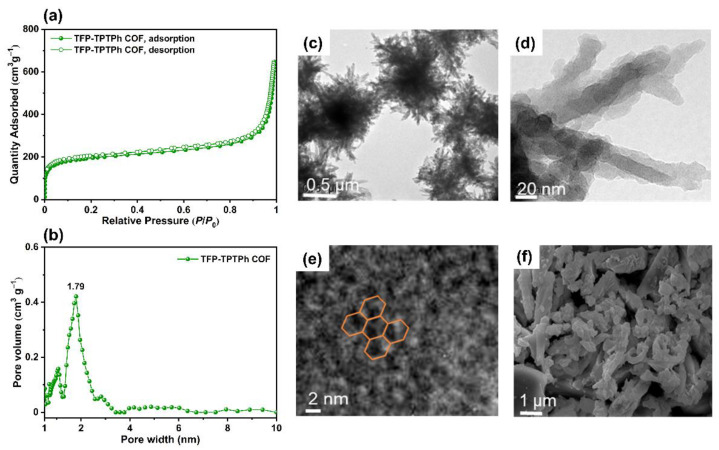
(**a**) N_2_ sorption isotherm recorded at 77 K of TFP-TPTPh COF. (**b**) Pore size distribution of TFP-TPTPh COF. (**b**–**e**) TEM images of TFP-TPTPh COF. (**f**) FE-SEM images of TFP-TPTPh COF.

The surface area and pore size of adsorbents and photocatalytic materials are extremely important; therefore, we studied the porosity features of the TFP-TPTPh COF by performing the N_2_ sorption isothermal analysis at 77 K (Figure 2a,b). The nitrogen sorption isotherm of the TFP-TPTPh COF revealed a type I isotherm, with an accelerated N_2_ uptake at lower pressure (*P*/*P*_0_ > 0.05), pursued by unmemorable N_2_ uptake in the pressure range (0.05 < *P*/*P*_0_ < 0.85) and a high N_2_ uptake in the pressure range higher (*P*/*P*_0_ > 0.85) (Figure 2a). This sorption behavior of TFP-TPTPh COF confirmed its microporous structure. Moreover, we used the Brunauer–Emmett–Teller (BET) model to investigate the surface area and pore volume of the TFP-TPTPh COF, indicating that TFP-TPTPh COF had a surface area of 724 m^2^ g^−1^ with a pore volume of 1.09 cm^3^ g^−1^ (Table S2). Furthermore, the pore size distribution profile based on the NLDF theory clarified the microporous architecture of TFP-TPTPh COF. Figure 2b and Table S2 show that TFP-TPTPh COF had a pore size of 1.79 nm. To gain more knowledge about the crystallinity of the TFP-TPTPh COF, we recorded its powder X-ray diffraction (PXRD) pattern (Figure S8). This pattern showed that the TFP-TPTPh COF had a hexagonal network connection with lengthy architecture. Figure S9 reveals that the most intense peak at 2*θ* = 3.91°, which we assign to the (100) facet of the lattice for TFP-TPTPh COF. The other minor diffraction peaks of at 2*θ* of at 7.45°, 7.78°, 11.76°, and 21.84° were delegated to the (210), (200), (310), and (001) facets, respectively. Based on Bragg’s law, the *d*_100_ spacing can predict the center-to-center spacing between two pores in a diagonal position. In Table S2, the *d*_100_ spacing of TFP-TPTPh COF corresponded to 2.26 nm. In addition, the 2*θ* of (001) could determine the interlayer spacing; for TFP-TPTPh COF, the 2*θ* value of (001) was 21.84°, and the interlayer distance was obtained as 4.06 Å (Table S2). We observed the morphology of the as-synthesized TFP-TPTPh COF using field-emission scanning electron microscopy (FE-SEM) and transmission electron microscopy (TEM). After solvent exfoliation in ethanol, the TEM images confirmed the assembly of the as-synthesized TFP-TPTPh COF into a considerable number of long rods with lengths made of several micrometers, and such micro-rods were tied by their mesoporous sidewalls (Figure 2c,d). The hexagonal polycrystalline state of the as-synthesized TFP-TPTPh COF was noticeable in the low-magnification TEM image (Figure 2e), which is comparable to the geometric lattice determined by PXRD. It has been reported that the degree of planarity of the building monomer can significantly affect the morphology of the resultant COF [46]. We also documented that the planar building monomer used to construct COFs frequently results in the formation of tubes, rods, or fibers [46,61]. Therefore, the high planarity of the TPTPh-3NH_2_ building monomer is thought to have contributed to the formation of the rod morphology of the TFP-TPTPh COF. On the other hand, the FE-SEM images exhibited that the TFP-TPTPh COF possessed an aggregated micro-rod with a smooth surface (Figure 2f); it also confirmed that the diameter and length of the as-synthesized COF were estimated to be 620 ± 30 nm and 5 ± 0.5 μm, respectively.

### 3.3. Photophysical and Photoelectric Properties of π-Electron-Extended Triazine-Based COF

**Figure 3:**
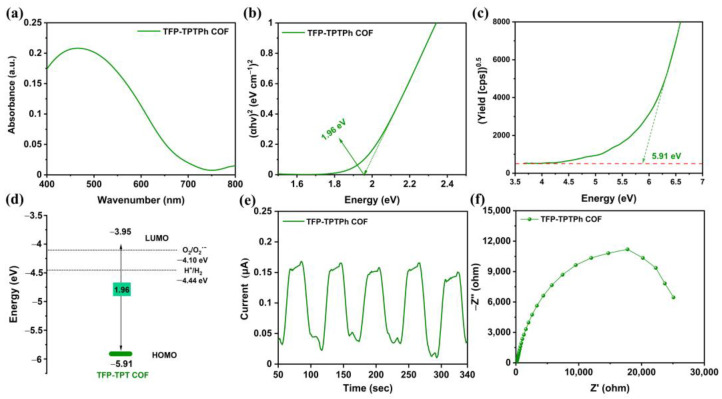
(**a**) UV–Vis DRS spectrum of TFP-TPTPh COF. (**b**) Tauc plot of TFP-TPTPh COF. (**c**) Photoelectron spectrum of TFP-TPTPh COF. (**d**) Electronic band level of TFP-TPTPh COF. (**e**) Periodic on/off photocurrent response of TFP-TPTPh COF. (**f**) EIS spectrum of TFP-TPTPh COF. All measurements were carried out at 25 °C.

The physicochemical characteristics of light-absorbing polymers determine their capacity to both absorb light and carry out the photocatalytic reaction [15,62,63]. In addition, due to their modulated π-electron delocalization and density, triazine derivatives have a solid reputation as fluorescent materials [64,65]. Triazine derivatives have attracted the attention of researchers in a wide range of photo-based applications, including organic light-emitting diodes, solar cells, and photocatalysts [66,67,68]. As a result of the unique electronic and optical features of triazine units, along with the elongated π-electron-delocalized skeleton arising from the inclusion of a phenyl ring, we investigated the photophysical and photoelectric features of the as-synthesized TFP-TPTPh COF by measuring its solid-state UV–Vis diffuse reflectance spectroscopy (UV–Vis DRS), photoluminescence (PL) emission, transient photocurrent measurements, and electrochemical impedance spectroscopy (EIS). UV–Vis DRS spectrum of the TFP-TPTPh COF revealed visible light absorption with a maximum absorption intensity at 518 nm and absorption borderlines prolonging into the near-infrared ambit (Figure 3a). Such a feature suggested that TFP-TPTPh COF could absorb a significant amount of visible radiation for photocatalytic reactions. The optical band gap for TFP-TPTPh COF, computed using the Tauc plot, was estimated to be 1.96 eV (Figure 3b and Table S3). Due to the triazine unit and the extended π-electron-delocalized framework, which improves the planarity of the COF skeleton and reduces the bandgap, TFP-TPTPh COF has a low bandgap [68]. A photoelectron spectrometer was applied to assess the energy level of the highest occupied molecular orbitals (HOMO) of the as-synthesized TFP-TPTPh COF (Figure 3c). The HOMO energy level was −5.91 eV for TFP-TPTPh COF (Figure 3c). The LUMO was calculated using the below equation (Equation (1)) to be −3.95 eV (Figure 3d and Table S3).

*E*_LUMO_ = *E*_HOMO_ + *E*_g_ (1)

The production and migration of photoinduced holes and electrons are crucial steps in photocatalytic reactions. Therefore, the PL spectrum examined how well the charge carrier generation and transfer performed. The as-synthesized TFP-TPTPh COF demonstrated an elevated PL emission maximum of around 525 nm in its PL emission spectrum (Figure S9). In addition, the generated electrons within the photocatalyst can be directly measured by the photocurrent. Therefore, we measured the photocurrent density of the as-synthesized TFP-TPTPh COF using a standard three-electrode instrument and under ultraviolet and visible light irradiation. Figure 3e reveals that the TFP-TPTPh COF had a transient photocurrent as the light of the solar simulator was on. The photocurrent response was carried out in five on/off cycles (per the 30 s). Furthermore, the EIS of as-synthesized TFP-TPTPh COF was investigated to evaluate its charge migration and charge carrier recombination properties. Figure 3f shows that the EIS spectrum of the TFP-TPTPh COF presented a typical semicircle Nyquist plot at high frequencies, revealing low interfacial impedances and high permeability conductivities for electrolyte ions. To better understand the conductivities of the intrinsic ohmic resistances, the intercept of the Z′ axis in the region of high frequencies was evaluated (*R*s). The *R*s value of the TFP-TPTPh COF was 58 Ω. These findings explained a lower resistance of TFP-TPTPh COF, due to its elongated π-electron-delocalized framework.

### 3.4. Dye Adsorption of π-Electron-Extended Triazine-Based COF

**Figure 4:**
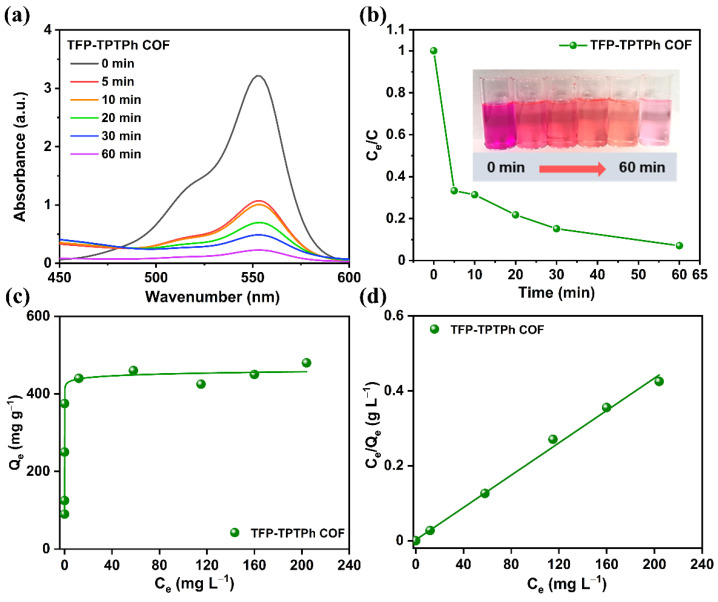
(**a**) UV–Vis spectra of an aqueous RhB solution (10 mL, 18 mg L^−1^) at various time intervals (from 0 to 60 min) after the addition of TFP-TPTPh COF (4 mg), all measurements were carried out at 25 °C, stirring speed = 800 rpm, pH = 7. (**b**) Rates of adsorption of RhB (10 mL, 18 mg L^−1^) onto the TFP-TPTPh COF (4 mg), all measurements were carried out at 25 °C, stirring speed = 800 rpm, pH = 7. (**c**) Langmuir isothermal model and (**d**) adsorption isothermal curve for the adsorption of RhB (10 mL, from 12.5 to 200 mg L^−1^) onto TFP-TPTPh COF (4 mg), all measurements were carried out at 25 °C, stirring speed = 800 rpm, stirring time = 24 h, pH = 7.

We anticipate that our TFP-TPTPh COF will be a promising adsorbent, due to its high surface area, appropriate pore size, and elongated π-electron-delocalized framework. Therefore, the cationic dye rhodamine B (RhB) was employed as the standard adsorbate to evaluate the adsorption performance of the TFP-TPTPh COF adsorbent [69]. We studied the physical adsorption behavior of the TFP-TPTPh COF by observing the adsorption variability of RhB at a maximum wavelength of 554 nm at different intervals (from zero to 60 min) after adding the TFP-TPTPh COF. Figure 4a shows that the addition of a sorbent quantity of TFP-TPTPh COF (4 mg) to an aqueous RhB solution (10 mL, 18 mg L^−1^) led to the complete adsorption of RhB dye molecules within 60 min. The removal efficiencies of RhB were nearly 84.84 and 94.5% within 30 and 60 min for TFP-TPTPh COF, respectively (Figure 4b), proving that our COF had a quite good adsorption capability for removing RhB organic dye. The adsorption of adsorbate on the adsorbent surface is time dependent. Adsorption isotherms define the adsorbate-adsorbent relationship when the adsorption process achieves equilibrium. Adsorption Langmuir kinetic isotherms are frequently employed to define the relationship between the concentration of adsorbed dye and the solution concentration at equilibrium [70,71]. The adsorption kinetic data were fitted to a Langmuir isotherm model to investigate the adsorption behavior of RhB organic dye on the surface of the TFP-TPTPh COF. With a correlation coefficient (R_L_^2^) of 0.9961 for TFP-TPT COF, Figure 4c represents that the Langmuir isothermal model had a good linear fit (*C*_e_/*Q*_e_ with respect to *C*_e_). The maximum adsorption capacity (*Q*_m_) of TFP-TPTPh COF was 480 mg g^−1^, which ranks among the greatest *Q*_m_ values for porous polymers ever to be recorded (Table S4 and Figure 4d). There are several factors that strongly affect the adsorption performance of the adsorbents, including surface area, π-π stacking interaction between the adsorbent and dye molecules, and porosities [72]. For surface area, it has been reported that the effectiveness of organic dye removal increases with adsorbent surface area. For π-π stacking interaction, we documented that expanding the π-electron delocalization system in the adsorbent improves its adsorption performance [15]. For porosity, it has been demonstrated that the dye was effectively absorbed by adsorbent materials with pores that matched the molecular size of the organic dye [15,73]. The pore size of the TFP-TPTPh COF is 1.79 nm, which is close to the molecular size of RhB (1.59 × 1.18 × 0.56 nm). Therefore, the high surface area, elongated π-electron-delocalized framework, and porosity of the TFP-TPTPh COF may account for its high adsorption performance. We investigated the recyclability of TFP-TPTPh COF in an aqueous solution of RhB using cyclic adsorption/regeneration tests. According to Figure S10, when the number of regeneration cycles was increased, the adsorption efficiency of the TFP-TPTPh COF slightly decreased, suggesting that TFP-TPTPh COF might be used as effective adsorbents for the removal of organic dyes from wastewater.

### 3.5. Photocatalytic Degradation of RhB over the π-Electron-Extended Triazine-Based COF

**Figure 5:**
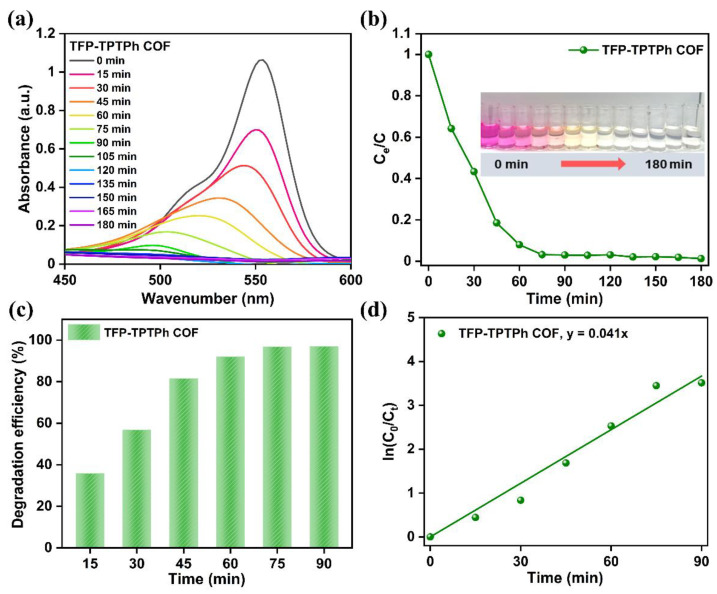
(**a**) UV–Vis spectra for the photodegradation of an aqueous RhB solution (56 mL, 10 mg L^−1^) at various time intervals (from 0 to 180 min) after the addition of TFP-TPTPh COF (7 mg), all measurements were carried out at 25 °C, stirring speed = 600 rpm, pH = 7. (**b**) Photocatalytic efficacy of TFP-TPTPh COF (7 mg) for the degradation of photodegradation of an aqueous RhB solution (56 mL, 10 mg L^−1^) under ultraviolet and visible light irradiation, all measurements were carried out at 25 °C, stirring speed = 600 rpm, pH = 7. (**c**) Pseudo-first-order kinetic curve for the degradation of photodegradation of an aqueous RhB solution. (**d**) Photodegradation efficiency for the adsorption of RhB (56 mL, 10 mg L^−1^) onto TFP-TPTPh COF (7 mg), all measurements were carried out at 25 °C, stirring speed = 600 rpm, pH = 7.

We evaluated the photocatalytic activity of TFP-TPTPh COF for the degradation of RhB under exposure to ultraviolet and visible light, taking into account its high surface area, porosity, elongated π-electron-delocalized framework, and narrow band gap. First, a UV–Vis spectroscopy of an aqueous environment of RhB (10 mg L^−1^, 56 mL), as a control test, was measured in the absence of a TFP-TPTPh COF photocatalyst. Figure S11 verifies that the degradation of such dye was challenging to carry out without such a photocatalyst under ultraviolet and visible light irradiation (Figure S11). Then, for photodegrading of RhB in water over TFP-TPTPh COF, the photodegradation performance of this COF (7 mg) was evaluated using an aqueous solution of RhB (10 mg L^−1^, 56 mL). Figure 5a,b shows that the dye molecules over the TFP-TPTPh COF photocatalyst were fully degraded within 75 min under ultraviolet and visible light. For assessing the photodegradation efficiency of RhB over TFP TFP-TPTPh COF photocatalyst, we used the following equation (Equation (2)) and plotted the graph of photodegradation efficiency versus different radiation times (Figure 5c).

(2) $$\mathrm{Photodegradation}\;\mathrm{efficiency}\;(\%)=\frac{C_{0}-C_{e}}{C_{0}}\times 100\;$$

where *C*_0_ was the initial concentration of the organic dye (mg L^−1^), and *C_e_* was the equilibrium concentration of the organic dye (mg L^−1^) at different irradiation times [15].

The photodegradation efficiency of RhB in water attained 92.03, 96.82, and 97.02% within 60, 75, and 90 min, respectively, of starting the ultraviolet and visible irradiation. This expressed that the TFP TFP-TPTPh COF photocatalyst had an excellent performance of photocatalytic activities for RhB degradation in water under ultraviolet and visible light. This high potential of TFP TFP-TPTPh COF photocatalyst for dye photodegradation could also be attributed to the E_LUMO_ of photocatalyst (−3.95 eV) being close to the reduction potential for superoxide radicals (O_2_˙^−^) (−4.10 Ev) Figure 5c. Under comparable photocatalytic conditions, our TFP TFP-TPTPh COF demonstrated an RhB degradation rate similar to, and in several cases higher than, that of a number of newly identified porous polymers (Table S5). To illustrate the degradation reaction kinetic of our TFP TFP-TPTPh COF photocatalyst, we fitted the kinetic data of the photocatalytic response according to the Langmuir–Hinshelwood kinetic model (Equation (3)).

(3) $$\ln\left(\frac{C_{0}}{C_{t}}\right)=kt\;\;$$

*C_t_* represents the final concentration in the solution of the organic dye (mg L^−1^), *k* is the pseudo-first-order rate constant, and *t* is irradiation time, respectively [74].

The degradation process induced by TFP TFP-TPTPh COF photocatalyst is shown in Figure 5d as pseudo-first-order reaction kinetic. In addition, our TFP-TPTPh COF photocatalyst displayed photocatalytic solid performance in the degradation of RhB, along with a reaction rate constant (4.1 × 10^−2^ min^−1^), indicating that TFP-TPT COF is a potential photocatalyst for removing RhB dye under ultraviolet–visible light irradiation. We measured the reusability of TFP-TPTPh COF photocatalyst in the degradation of RhB through three cycles to ensure its suitability for economic applications. Figure S12 reveals that the TFP-TPTPh COF photocatalyst exhibited superior photocatalytic stability throughout its recycling and high photocatalytic activity, up to 96%, after the third recycling.

During photocatalytic degradation of organic dye, photogenerated holes (h^+^), superoxide radicals (O_2_˙^−^), and singlet oxygens (^1^O_2_) are generally reactive species. Therefore, to explore the mechanism of the photocatalytic reaction of TFP-TPTPh COF photocatalyst, we studied the influence of different charge carrier scavengers (ethylenediaminetetraacetic acid disodium salt (EDTA-2Na) for holes (h^+^), benzoquinone (BQ) for (O_2_˙^−^), and sodium azide (NaN_3_) for (^1^O_2_)) on the photodegradation reliability of RhB. Figure S13 shows that the addition of NaN_3_ or EDTA-2Na to the RhB solution resulted in only a slight decrease in degradation efficiency. At the same time, the lack of O_2_˙^−^ and the existence of BQ led to a substantial decline in photocatalytic activity. These findings confirmed that the TFP-TPTPh COF photocatalyst is an excellent photocatalyst for generating O_2_˙^−^, which is produced when electrons react with oxygen dissolved in water.

### 3.6. Photocatalytic Hydrogen Evolution from Water

**Figure 6:**
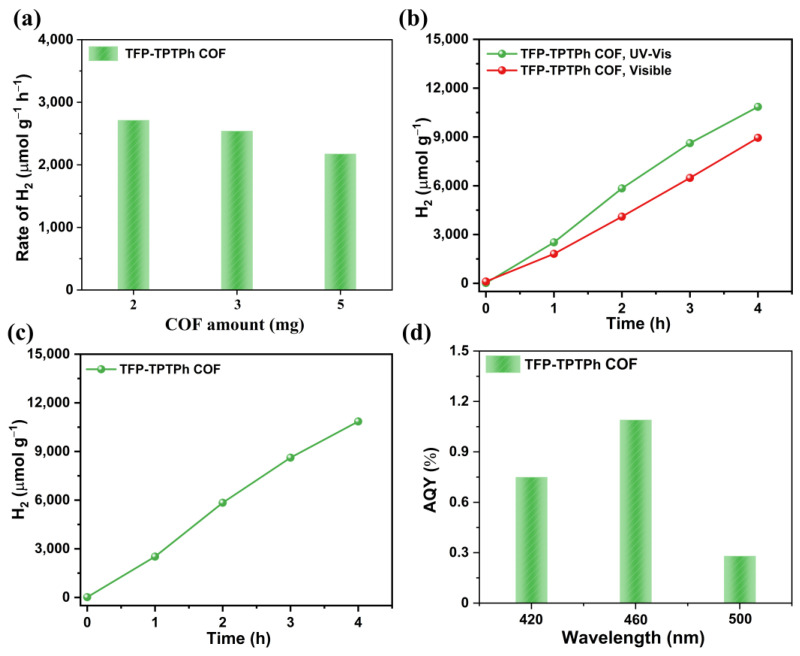
(**a**) Effect of TFP-TPTPh COF photocatalyst amount on the hydrogen production activity. (**b**) Effect of illumination source on the hydrogen production activity. (**c**) Hydrogen production activity over four hours of using TFP-TPTPh COF photocatalyst under ultraviolet and visible light irradiation. (**d**) AQYs of the TFP-TPTPh COF photocatalyst that measured at different wavelengths of light.

We evaluated TFP-TPTPh COF photocatalyst for H_2_ evolution from water at 25 °C for four hours that used a Xe lamp as an ultraviolet and visible illumination source. In a Pyrex photoreactor with a suitable sacrificial electron donor (SED), the TFP-TPTPh COF photocatalyst was dispersed in H_2_O/DMF (9:1 *v/v* %) co-solvent to establish a photocatalytic system. The temperature of the photocatalytic system was maintained at 25 °C by using flowing water, and a gas chromatograph (GC) was used to monitor and record the produced gas from the system once every hour. Several parameters were investigated, including the SED, amount of photocatalyst, and illumination source. To determine the best SED, we measured the hydrogen evolution rate (HER) in solutions containing ascorbic acid (AA), triethanolamine (TEOA), and triethylamine (TEA). Figure S14 exhibits that the highest HER was obtained in the presence of AA; as a result, we selected AA for further studies. The protonation of N–H function units may have contributed to the greatest HER of the TFP-TPTPh COF photocatalyst utilizing AA. According to reports, the protonation of COF improves the hydrophilicity of COF and decreases its bandgap, which causes it to absorb a considerable amount of light [75,76]. The photocatalyst mass has been shown to seriously influence photocatalytic performance, due to saturation light absorption when an adequate amount of photocatalyst is added [77,78,79]. Consequently, the influence of photocatalyst mass on the photocatalytic HER was examined by changing the mass (from 2.0 to 5.0 mg). The best COF performance was attained with 2.0 mg of TFP-TPTPh COF photocatalyst (Figure 6a and Figure S15). As a result, we selected a photocatalyst mass of 2.0 mg for further studies. The illumination source was also studied using visible and full (ultraviolet and visible) illumination sources. Figure 6b exhibits that the obtained photocatalytic HER under ultraviolet and visible illumination is slightly higher than that under visible illumination. As a result, we used ultraviolet and visible illumination for further studies. Through the optimized photocatalytic reaction, the amount of H_2_ increased with increasing the irradiation time and reached approximately 10,849 μmol g^−1^ after four hours (Figure 6c). Furthermore, our TFP-TPTPh COF photocatalyst exhibited an HER efficiency of 2712 μmol g^−1^ h^−1^. This high HER efficiency of TFP-TPTPh COF could be attributed to its high surface area, elongated π-electron-delocalized framework, and suitable band gap.

Overall, our TFP-TPTPh COF photocatalyst exhibited excellent photocatalytic performance for the reduction of H_2_O to produce H_2_, without the need for additional co-catalysts. Table S6 provides an overview of the HERs of several photocatalytic polymeric materials; our COF had comparable, and in several cases higher, photocatalytic efficiency. The results of the control photocatalytic study proved that COF is required for photocatalytic hydrogen activity because no hydrogen could be detected in pure distilled water in the absence of a COF photocatalyst (Figure S16). The apparent quantum yield (AQY) for producing hydrogen was tested as a variable of light source wavelength using bandpass filters with wavelengths of 420, 460, and 500 nm to evaluate the photocatalytic activities of the TFP-TPTPh COF across the wavelength spectrum. Figure 6d reveals that the AQYs of TFP-TPTPh COF photocatalyst were 0.75, 1.09, and 0.28% at 420, 460, and 500 nm, respectively, which matches with the absorption spectrum of the TFP-TPTPh COF. After measuring the photocatalytic H_2_ evolution, the FTIR analysis of the recovered TFP-TPTPh COF was used to examine the structural stability of a photocatalyst. The remarkable structural stability of our TFP-TPTPh COF was demonstrated in Figure S17, which showed no discernible alterations of the COF before or after the photocatalytic processes.

## 4. Conclusions

In summary, a π-electron-extended triazine-based COF (TFP-TPTPh COF) was synthesized through the polycondensation reaction of TPTPh-3NH_2_ and TFP-3OHCHO in a mixed solvent at 120 °C for three days in the presence of acetic acid (6 M). FTIR and solid-state ^13^C NMR (^13^C SS-NMR) spectroscopies were used to confirm the molecular structure of the triazine-based COF. The TGA, BET, and PXRD measurements revealed that triazine-based COF had massive thermal stability with a char yield of 63.41%, a high surface area of 724 m^2^ g^−1^, and outstanding crystallinity. The triazine-based COF also exhibited a narrow band gap of 1.96 eV, indicating its effective photophysical and photoelectric properties. In addition, we tested the applicability of our triazine-based COF for the removal and photocatalytic degradation of RhB dye molecules, as well as the photocatalytic activity of hydrogen production from water. The triazine-based COF performed well in the adsorption and photocatalytic degradation of the RhB, with a *Q*_m_ of 480 mg g^−1^ and a photocatalytic degradation reaction rate constant of 4.1 × 10^−2^ min^−1^. In addition, the triazine-based COF showed a great capacity for producing H_2_ from water with an HER of 2712 μmol g^−1^ h^−1^ of under ultraviolet–visible light irradiation without needing for a co-catalyst. The triazine-based COF also showed an AQY of 1.09% at 460 nm. Thus, our COF offers the chance to develop innovative organic photocatalyst, which could be used to remove and photo-catalytically degrade RhB dye from water, as well as potentially generate H_2_ from water.

## Data Availability

Data supporting the findings of this study are available on request by the corresponding author.

## Supplementary Materials

The following supporting information can be downloaded at: https://www.mdpi.com/article/10.3390/polym15071685/s1, Scheme S1: Synthesis of TPTPh-3NH_2_; Scheme S2: Synthesis of 1,3,5-triformylphloroglucinol (TFP-3OHCHO); Figure S1: IR spectrum of TPTPh-3NH_2_; Figure S2: ^1^H-NMR spectrum of TPTPh-3NH_2_; Figure S3: ^13^C-NMR spectrum of TPTPh-3NH_2_; Figure S4: IR spectrum of 1,3,5-triformylphloroglucinol (TFP-3OHCHO); Figure S5: ^1^H-NMR spectrum of 1,3,5-triformylphloroglucinol (TFP-3OHCHO); Figure S6: ^13^C-NMR spectrum of 1,3,5-triformylphloroglucinol (TFP-3OHCHO); Figure S7: TGA analysis of TFP-TPTPh COF; Figure S8: PXRD pattern of TFP-TPTPh COF; Figure S9: Fluorescence spectrum of TFP-TPTPh COF; Figure S10: Reusability of TFP-TPTPh COF for the removal of RhB within 60 min; Figure S11: (a) UV–Vis spectra and (b) Photocatalytic efficacy of the control experiment of RhB upon UV and visible light irradiation without catalyst; Figure S12: Reusability of TFP-TPTPh COF for the photodegrading of RhB; Figure S13: Effect of different scavengers, NaN_3_, BQ, and EDTA-2Na on the photocatalytic degradation of RhB (10 mg L^−1^) by TFP-TPTPh COF under UV and visible light irradiation; Figure S14: Effect of sacrificial electron donors the hydrogen production; Figure S15: Effect of TFP-TPTPh COF photocatalyst amount on the hydrogen production activity; Figure S16: Control experiment of the TFP-TPTPh COF; Figure S17: FTIR spectra of TFP-TPTPh COF before and after the photocatalytic H_2_ evolution measurement; Table S1: Values of T_d10%_, and Char yield of TFP-TPTPh COF; Table S2: BET and PXRD parameters of TFP-TPTPh COF; Table S3: Absorption maxima and energy levels of the TFP-TPTPh COF; Table S4: Maximum adsorption capacities of RhB on the TFP-TPTPh COF, compared with those of other reported materials; Table S5: Photodegradation performance of RhB on the TFP-TPTPh COF, compared with those of other reported materials; Table S6: HERs of TFP-TPTPh COF, compared with those of other reported materials [80,81,82,83,84,85,86,87,88,89,90,91,92,93,94,95,96,97,98,99,100,101,102].
