# Supplementary material for: π-Electron-Extended Triazine-Based Covalent Organic Framework as Photocatalyst for Organic Pollution Degradation and H2 Production from Water
Source: Polymers (Basel). 2023 Mar 28;15(7):1685. doi: 10.3390/polym15071685 (PMC10096642; doi:10.3390/polym15071685)
Supplement: Supplementary file 1 [file polymers-15-01685-s001.zip › polymers-2287520-supplementary.pdf]

Supplementary Materials

Characterizations

Fourier-transform infrared (FRIR) spectroscopy was recorded using a Bruker Tensor 27 FTIR spectrophotometer (Billerica, MA, USA) and the conventional KBr plate method; 32 scans were collected at a resolution of 4 cm^{-1}. Solid state nuclear magnetic resonance (SSNMR) spectroscopy was recorded using a Bruker Avance 400 NMR spectrometer and a Bruker magic-angle-spinning (MAS) probe (Billerica, MA, USA), running 32,000 scans. Thermogravimetric analysis (TGA) was performed using a TA Q-50 analyzer (TA Instruments, New Castle, DE, USA) under a flow of N_2. The samples were sealed in a Pt cell and heated from 40 to 700 °C at a heating rate of 20 °C min^{-1} under N_2 at a flow rate of 50 mL min^{-1}. Nitrogen adsorption-desorption measurements were carried out using a BelSorp max instrument (Microtrac MRB, Montgomeryville, PA, USA). Before measuring gas adsorption, the as-prepared samples (50 mg) were washed with anhydrous tetrahydrofurane for 24 h using Soxhlet extraction. The solvent was filtered, and the samples were activated for 10 h under pressure at 150 °C. The samples were then used for gas adsorption-desorption measurements at 77 K from 0 to 1 atm. Their specific surface areas were calculated using the Brunauer-Emmett-Teller (BET) methodology. The pore distributions were calculated from the sorption data using the quenched solid state density functional theory. Field-emission scanning electron microscopy (FE-SEM) was conducted using a JEOL JSM-7610F scanning electron microscope (JEOL Ltd., New England, MA, USA). Samples were subjected to Pt sputtering for 100 s prior to observation. Transmission electron microscopy (TEM) was performed using a JEOL-2100 scanning electron microscope (JEOL Ltd., New England, MA, USA), operated at 200 kV. Samples for UV–Vis and fluorescence spectroscopy were dissolved in suitable organic solvents and placed in a small quartz cell (0.2 × 1.0 × 4.5 cm^3). UV–Vis–NIR spectra were recorded at 25 °C using a Jasco V-570 spectrometer (Jasco, Hachioji, Japan), with deionized water as the solvent. Photoluminescence (PL) was measured by HITACHI F-4500 (Tokyo, Japan), using 150W Xe Lamp. The samples were mixed with DMF then placed in a small quartz cell and excited with 360 nm wavelength. Ultraviolet Photoelectron Spectroscopy (UPS) was carried out with ESCA003500 (ULVAC-PHI, Inc., Kanagawa, Japan), using the intensity of He I (21.2 eV). The samples were dropped on indium tin oxide (ITO) glasses. The photodegradation of dye and photocatalytic hydrogen evolution were performed using solar simulator of 7IS0503A, SOFN Instruments Co. Ltd. (Beijing, China) and the yield analysis of photocatalytic hydrogen evolution was obtained with Nexis GC-2030 (Shimadzu, Japan).

Synthetic procedures

Synthesis of 4′,4′′′,4′′′′′-(1,3,5-triazine-2,4,6-triyl)tris(([1,1′-biphenyl]-4-amine)) (TPTPh-3NH_2)

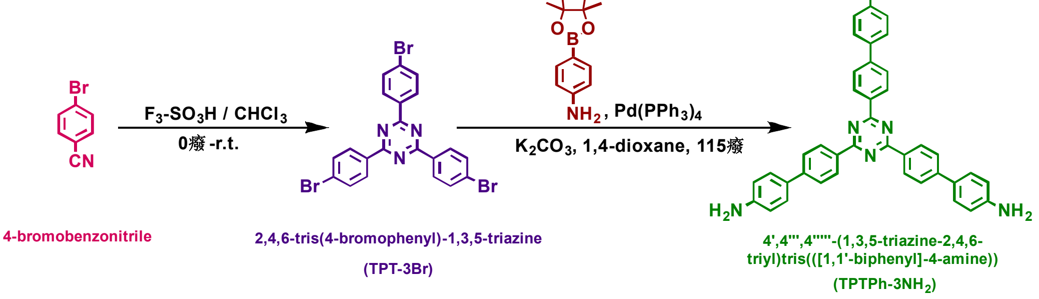

Scheme S1. Synthesis of TPTPh-3NH_2.

2,4,6-tris(4-bromopheny)-1,3,5-triazine (TPT-3Br): In a 100 mL two neck-bottle, 4-bromobenzonitrile (1.5 g, 8.24 mmol) in dry chloroform (20 mL) was cooled to 0 °C and afterward trifluoromethanesulfonic corrosive (4 mL, 0.045 mmol) was drop wisely added

under nitrogen atmosphere. The response mixture was mixed for 30 min at 0 °C and afterward warmed to room temperature. After stirring for 20 extra hours at room temperature, the mixture was added to ice-water (50 mL). The reaction solution was neutralized by sodium carbonate ($NaCO_3$). The product was collected by filtration and dried under vacuum overnight to get 2.4,6-tris (4-bromophenyl-1,3,5-triazine as a white solid. FT-IR (powder): 1578, 1518, 1400, 1372, 1173, 1069, 1011, 843, 806, 498 cm^{-1}. ^{1}H-NMR ($CDCl_3$, 25 °C, 500 MHz): 8.60 (d, *J* = 8.5 Hz, 6H), 7.70 (d, *J* = 8.5Hz, 6H). ^{13}C-NMR ($CDCl_3$, 25 °C, 125 MHz): 171.22, 134.91, 132.01, 130.48, 127.88.

4',4''',4'''''-(1,3,5-triazine-2,4,6-triyl)tris(([1,1'-biphenyl]-4-amine)) (TPTPh-3NH$_2$): In a 250 mL two neck-bottle and under nitrogen atmosphere, 2,4,6-tris(4-bromopheny)-1,3,5-triazine (TPT-3Br) (0.4 g, 0.73 mmol, 1 equivalent), 4-aminophenylboronic acid pinacol ester (0.97 g, 4.43 mmol, 6 equivalent), tetrakis(triphenylphosphine) palladium (Pd $(PPh_3)_4$, 86 mg, 0.073 mmol, 0.1 equivalent), and potassium carbonate (0.76 g, 5.5 mmol, 7.5 equivalent) were weighted. After the solids were evacuated under high pressure for 15 min, dioxane (30 mL) and water (7.5 mL) were added, and the reaction mixture was heated at 115 °C for 72 h. The reaction mixture was poured into ice-water (50 mL) and the precipitate was separated by filtration and washed several times with methanol to obtain TPT-3PhNH$_2$ as a green-brown powder (0.34 g, 80%). FT-IR (powder): 3446-3326, 1600, 1509 cm^{-1}, and 1281 cm^{-1}. ^{1}H-NMR (DMSO-d$_6$, 25 °C, 500 MHz): 8.71 (d, *J* = 9.0 Hz, 6H), 7.85 (d, *J* = 9.0 Hz, 6H), 7.56 (d, *J* = 9.0 Hz, 6H), 6.69 (d, *J* = 9.0 Hz, 6H), and 5.45 ppm. ^{13}C-NMR (DMSO-d$_6$, 25 °C, 125 MHz): 171.46, 150.21, 145.71, 133.37, 129.96, 128.38, 126.42, 126.03, and 114.79 ppm.

Synthesis of 1,3,5-triformylphloroglucinol (TFP-3OHCHO)

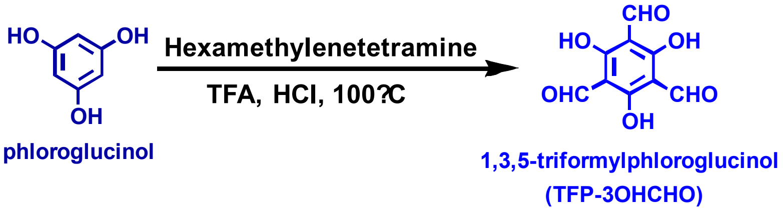

Scheme S2. Synthesis of 1,3,5-triformylphloroglucinol (TFP-3OHCHO).

Hexamethylenetetramine (5 g, 35.67 mmol) and phloroglucinol (2 g, 15.86 mmol) were added to a 250 mL two-neck round-bottomed flask with trifluoroacetic acid (30 mL) under N_2. The mixture solution was heated at 100 °C for 3 h. After adding 3 M hydrochloric acid (100 mL), the mixture was heated at 100 °C for 1 h. The reaction mixture was extracted three times with dichloromethane until it cooled down to room temperature. The extract was concentrated with rotatory evaporation and added to ethanol (50 mL). The product was filtered and washed completely with ethanol (20 mL). The solid was dried under vacuum to obtain 1,3,5-triformylphloroglucinol as a pink powder 0.6 g (18 %). FTIR (powder)： 3455, 2927, 2883, 2856, 1644, 1601, 1430, 1383, 1255, 1192, 964, 875, 786, 608 cm^{-1}. ^{1}H-NMR ($CDCl_3$, 25 °C, 500 MHz) ：δ = 14.10 (s, 3H), 10.14 (s, 3H) ppm. ^{13}C-NMR ($CDCl_3$, 25 °C, 125 MHz) ： δ = 192.06, 173.58, 102.88 ppm. FT-MS (EI-): m/z (%) = 209.009 [M-1].

Spectral Profiles of monomers

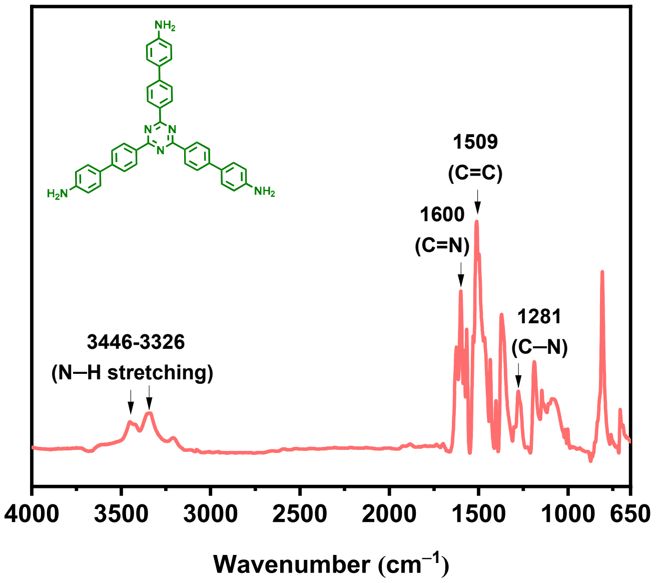

Figure S1. IR spectrum of TPTPh-3NH2.

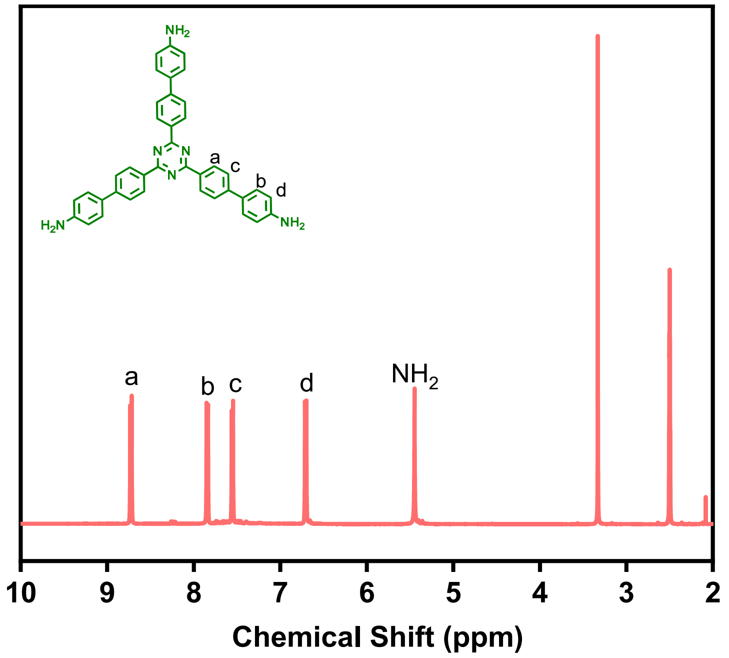

Figure S2. ^{1}H-NMR spectrum of TPTPh-3NH2.

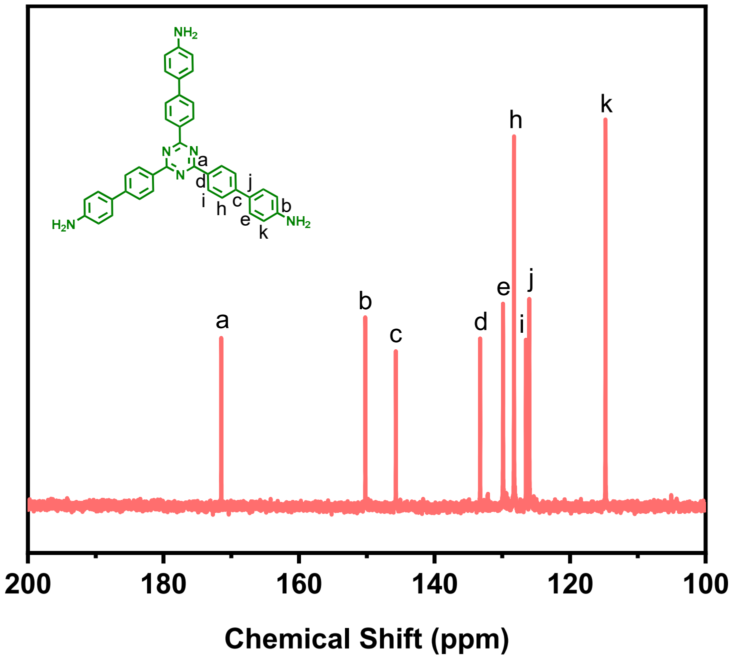

Figure S3. ^{13}C-NMR spectrum of TPTPh-3NH2.

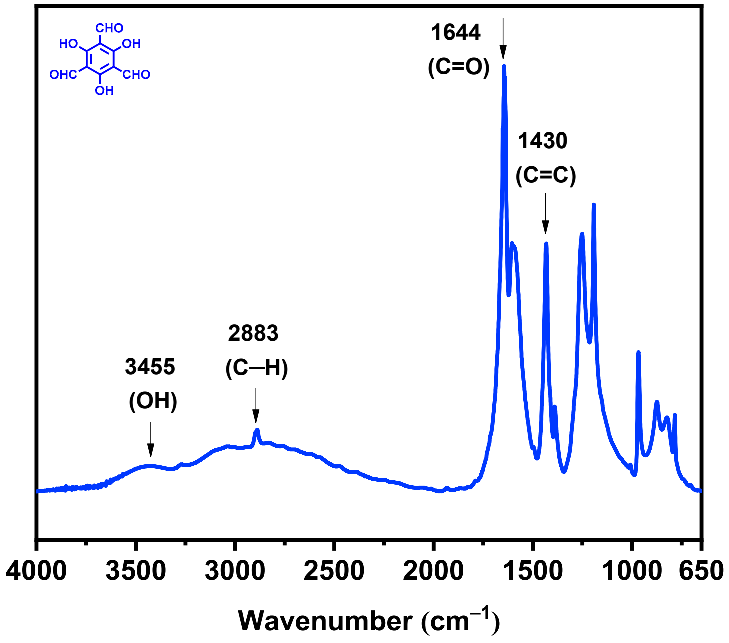

Figure S4. IR spectrum of 1,3,5-triformylphloroglucinol (TFP-3OHCHO).

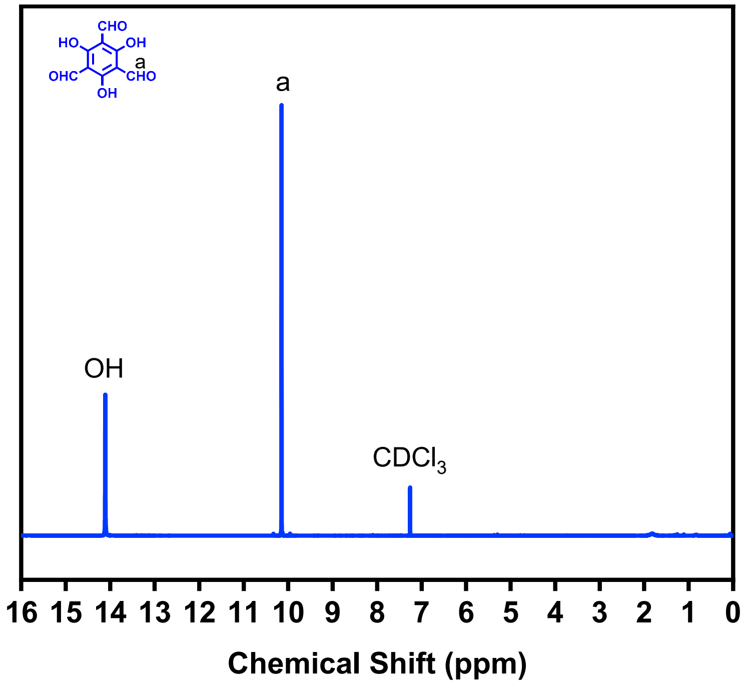

Figure S5. ^{1}H-NMR spectrum of 1,3,5-triformylphloroglucinol (TFP-3OHCHO).

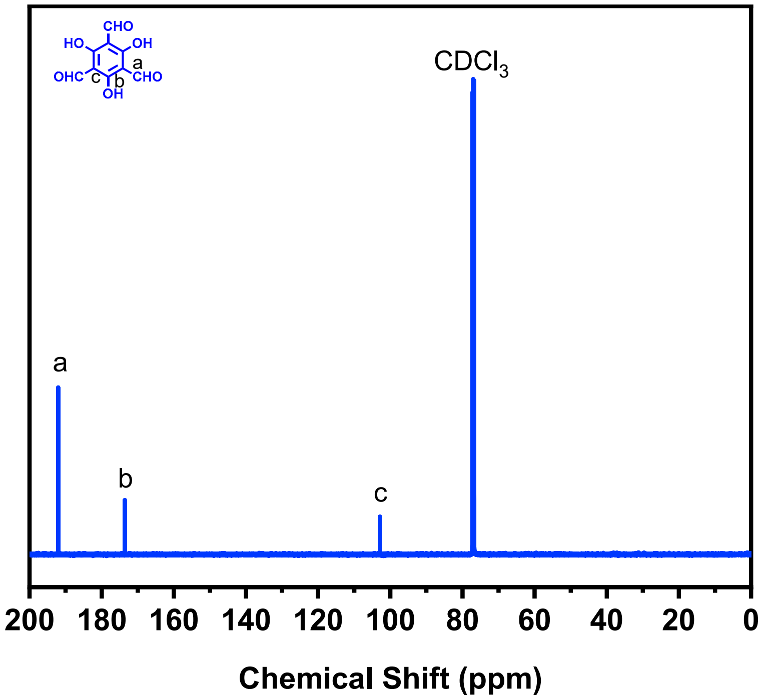

Figure S6. ^{13}C-NMR spectrum of 1,3,5-triformylphloroglucinol (TFP-3OHCHO).

Thermogravimetric analysis

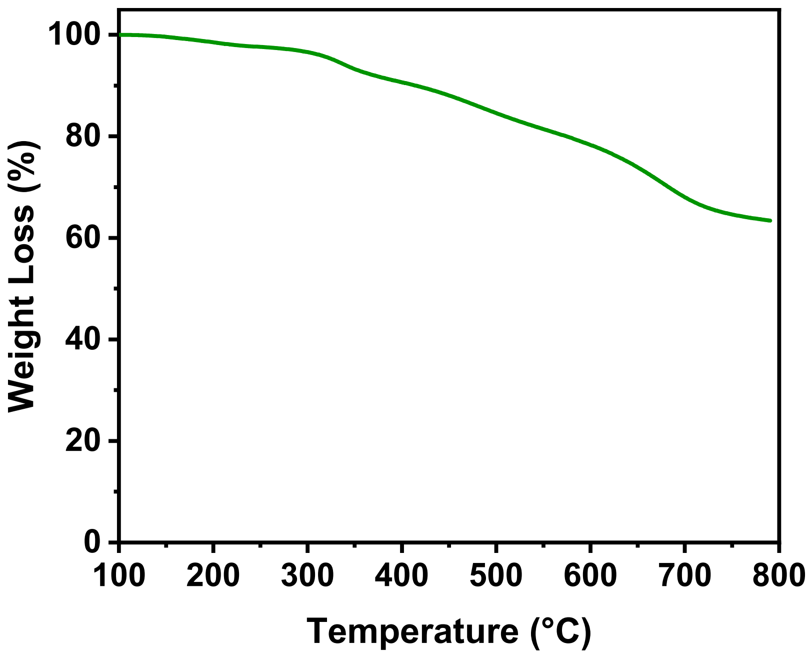

Figure S7. TGA analysis of TFP-TPTPh COF.

Table S1. Values of $T_{d10\%}$, and Char yield of TFP-TPTPh COF.

COF	$T_{d10\%}$ (°C)	Char yield (%)
TFP-TPTPh COF	414.33	63.41

BET parameter of COF

Table S2. BET and PXRD parameters of TFP-TPTPh COF.

COF	S_{BET} ($m^2\ g^{-1}$)	d_{110} (nm)	Interlayer distance (Å)	Pore Volume ($cm^3\ g^{-1}$)	Pore Size (nm)
TFP-TPTPh COF	724	2.26	4.06	1.09	1.79

Powder XRD analysis

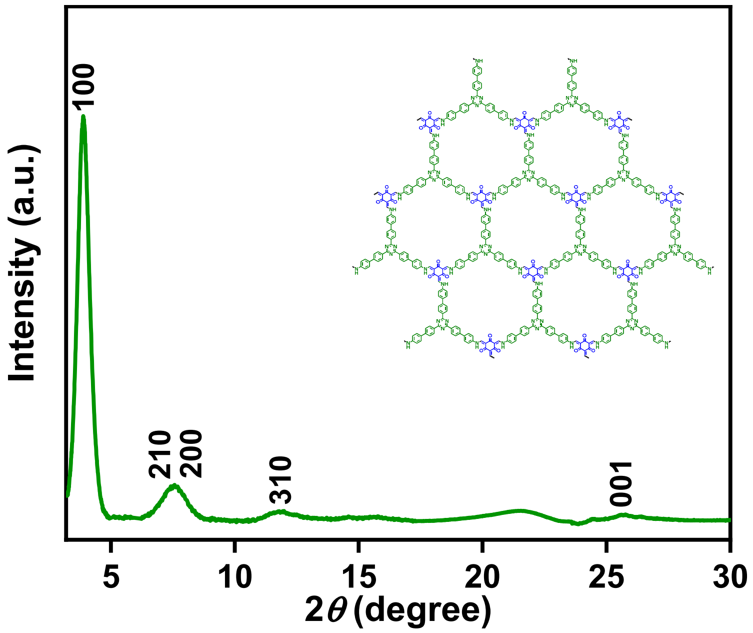

Figure S8. PXRD pattern of TFP-TPTPh COF.

Fluorescence spectroscopy

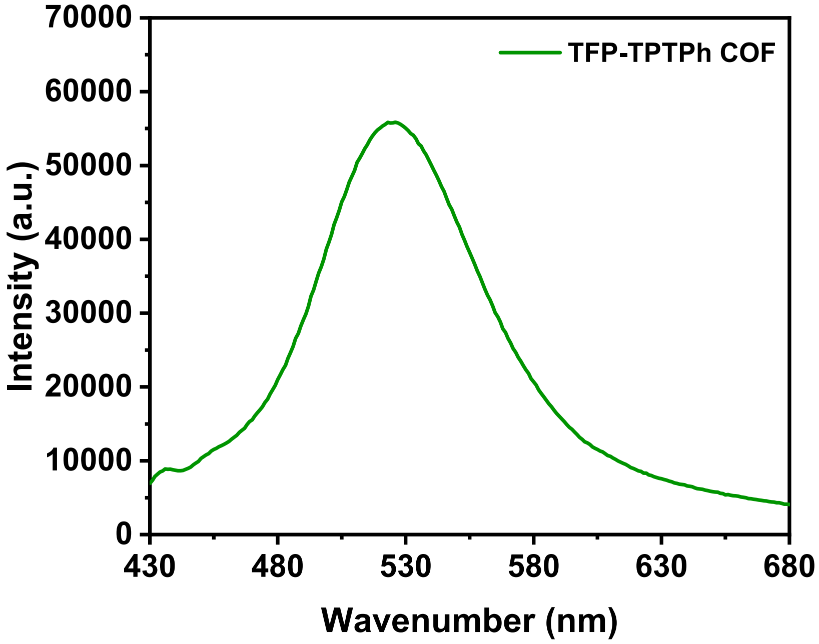

Figure S9. Fluorescence spectrum of TFP-TPTPh COF.

Electronic band levels

For optical band gaps for TFP-TPTPh COF, the Tauc plot was performed according to the Kubelka–Munk function [80,81]. The following equation is used to calculate optical band gap using absorption spectra:

$$\alpha h\nu = A(h\nu - Eg)^{n/2} \quad \text{(S1)}$$

Where α is the optical absorption coefficient, h is the Planck constant, ν is the light frequency, Eg is the optical band gap, and A the a cinstant depending on electron-hole mobility. The value of n is 1. Then, The Eg of the samples could well be determined from a plot of $(\alpha h\nu)^{1/2}$ versus energy (hν), and its value can be derived from the intercept of the tangent to the X axis.

For the energy level of the highest occupied molecular orbital (HOMO) of the as-prepared TFP-TPTPh COF, a photoelectron spectrometer was used [82,83]. The photoelectric effect is used in the photoelectron spectrometer method, which measures the photocurrent while irradiating the sample with monochromatic light of varying energy. There is a linear relationship between the nth power of photoelectron emission yield ($Y^{1/2}$) and the excitation photon energy (E) for semiconductors [84–86]. The common value of n photoelectron spectrometer is 0.5. This photoemission process is given by the following

$$Y^{1/2} \propto (E - HOMO) \quad \text{(S2)}$$

Therefore, to determine the HOMO level of our TFP-TPTPh COF, we plotted the squar root of the photoelectron emission yield versus the excitation photon energy. The threshold energy is equal the HOMO energy of the TFP-TPTPh COF.

Table S3. Absorption maxima and energy levels of the TFP-TPTPh COF.

Photocatalyst	λ_{max}[nm][a]	E_g[eV][b]	HOMO[eV][c]	LUMO[eV][d]
TFP-TPTPh	518	1.96	–5.91	–3.95

[a] Absorption was determined in solid state using reflectance mode; [b] Determined by the onset of UV-vis absorption; [c] Caliculated from the photoelectron spectrometer; [d] Calculated from LUMO = HOMO– Eg.

Photocurrent Measurement

The photocurrent response measurements were performed on a Zahner Zennium 6273E workstation equipped with visible-light irradiation with a conventional three-electrode cell including a Pt wire counter electrode, Ag/AgCl as reference electrode (3 M NaCl) and an indium tin oxide (ITO) glass as working electrode. About 5 mg of TFP-TPTPh COF were dispersed into an acetonitrile solution (1 mL) with 30 μL Nifion and sonicate for 30 min to obtain a slurry mixture. After that, 200 μL of as-prepared slurry was spread onto ITO glass with an active area of 6.875 cm^2. Here, 0.5 M Na_2SO_4 aqueous solution was prepared as electrolyte. 0.8 V constant potential was applied with the 20 s light on-off after a certain time interval to record the photo and dark current. A 300 W Xe lamp with a visible-light band-pass filter (λ > 450 nm) was employed as the excitation light source.

Dye adsorption Experiments

The performance of the TFP-TPTPh COF for dye removal from water was demonstrated using RhB organic dye. In this experiment, a weight of 4 mg of TFP-TPTPh COF was added to an aqueous solution of RhB (10 mL, 18 mg L^{-1}) in a glass vial with stirring for (0, 5, 10, 20, 30, and 60 min) at a rate of 800 rpm, at 25 °C and pH = 7. Centrifugation (6000 rpm, 10 min) was then carried out to isolate the supernatant, then the UV–Vis spectrum of the isolated supernatant was recorded.

Different concentrations of dye solution (from 12.5 to 200 mg L^{-1}) were used in order to obtain adsorption isothermal curves. At every experiment, a specific amount of TFP-TPTPh COF (4 mg) was added to the previous prepared RhB aqueous solutions (10 mL) in a glass vial with stirring at a rate of 800 rpm for a period of 24 h, at 25 °C and pH = 7. The clear dye solution was separated from mother liquor using centrifugation and then, its UV–Vis spectrum was measured. The equilibrium adsorption of dye per unit mass of the adsorbent, Q_e (mg g^{-1}) [87], was estimated as follows:

$$Q_e = (C_0 - C_e)\ x\ V\ x\ m^{-1} \tag{S3}$$

Where C_0 (mg L^{-1}) is the initial dye concentration in the liquid phase, C_e (mg L^{-1}) is the equilibrium dye concentration in the liquid phase, V (L) is volume of dye solution, and m (mg) is the mass of COF.

The adsorption isotherms were fitted using the Langmuir isothermal model (linear form) which presented as follows:

$$\frac{C_e}{Q_e} = \frac{1}{K_L Q_m} + \frac{C_e}{Q_m} \tag{S4}$$

where K_L (L mg^{-1}) is the Langmuir constant; and Q_m (mg g^{-1}) is the maximum equilibrium adsorption of dye per unit mass of the adsorbent.

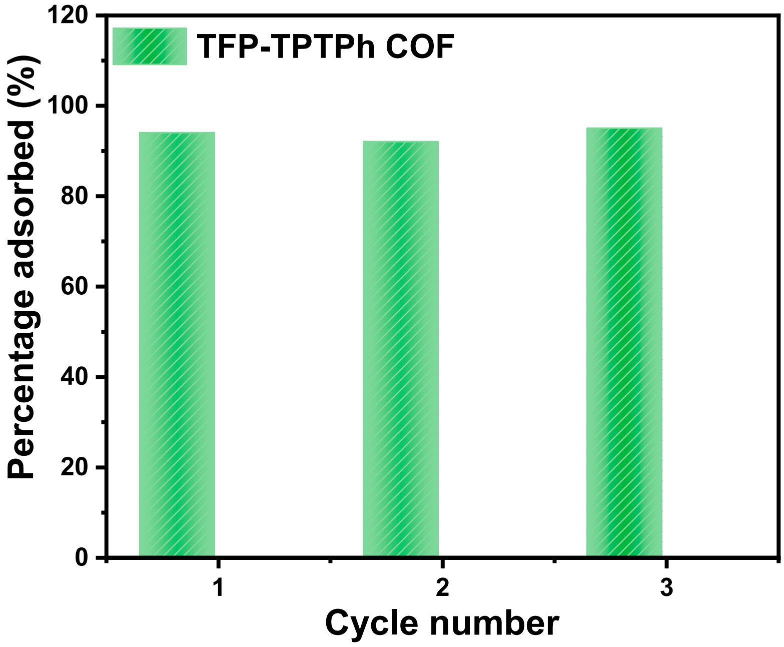

Figure S10. Reusability of TFP-TPTPh COF for the removal of RhB within 60 min.

Table S4. Maximum adsorption capacities of RhB on the TFP-TPTPh COF, compared with those of other reported materials.

Adsorbent	Dye	Q_m (mg g^{-1})	BET(m^2 g^{-1})	Amount/dye conc.	Ref.
CMP-YA	RhB	535	1410	0.2 mg mL^{-1}/25 mg L^{-1}	[88]
Py-BF-CMP	RhB	1905	1306	0.2 mg mL^{-1}/25 mg L^{-1}	[73]
TPE-BF-CMP	RhB	1024	777	0.2 mg mL^{-1}/25 mg L^{-1}	[73]
TPA-BF-CMP	RhB	926	590	0.2 mg mL^{-1}/25 mg L^{-1}	[73]
Ttba-TPDA-COF	RhB	833	726	0.5 mg mL^{-1}/20–800 mg L^{-1}	[89]
CuP-DMNDA-COF/Fe	RhB	378–429	273	0.25 mg mL^{-1}/16 mg L^{-1}	[90]
BIPE-BIPE	RhB	352	918	0.4 mg mL^{-1}/25 mg L^{-1}	[91]
BIPE-Py	RhB	1027	1400	0.4 mg mL^{-1}/25 mg L^{-1}	[91]
BIPE-TPT	RhB	739	903	0.4 mg mL^{-1}/25 mg L^{-1}	[91]
TFP-TPTPh COF	RhB	480	724	0.4 mg mL^{-1}/18 mg L^{-1}	**This work**

Photodegradation Experiments

The blank experiment without adding TPF-TPTPh COF was conducted with 56 mL of RhB solution (10 mg L^{-1}) and irradiated for 180 min. The distance between the liquid level and the filter is fixed at 8 cm.

In a typical Photodegradation experiment, a weight of 7 mg of TFP-TPTPh COF was added to an aqueous solution of RhB (56 mL, 10 mg L^{-1}) in a glass in a 100 mL sandwich beaker and magnetically stirred with the rate of 600 rpm for 60 min in the dark for reaching the saturation of adsorption of dye. The entire mixture was kept at room temperature by using circulating water. The entire mixture was performed irradiation during the photocatalytic experiment for 180 min for TFP-TPTPh COF. An aliquot of the reaction mixture (3 mL) was taken by a pipette every 30 min to monitor the degradation process, for which the catalyst was removed through centrifugation (4000 rpm, 2 min). Then, the UV–Vis spectrum of the isolated supernatant was recorded. The degradation efficiency (%) was calculated from equation listed below:

$$\text{Degradation efficiency (\%)} = \frac{C_0 - C_e}{C_0} \times 100 \quad (2)$$

Where C_0 (mg L^{-1}) is the initial dye concentration in the liquid phase and C_e (mg L^{-1}) is the equilibrium dye concentration in the liquid phase at t = 0 and t minutes of photocatalytic reaction.

For reusability of TFP-TPTPh COF for organic RhB dye degradation, the solutions were filtered to recover the TFP-TPTPh COF catalysts, and to guarantee that the adsorbed dyes were removed, the catalysts were washed with a significant amount of water and ethanol. The recovered catalysts were then activated under vacuum at 80 °C for 1 night. Each cycle experiment was carried out under the identical conditions to verify the correctness of the results. The radical scavenger experiments were performed using sodium azide (32 mg mL^{-1}), benzoquinone (54 mg mL^{-1}) and ethylenediaminetetraacetic acid disodium salt (186 mg mL^{-1}). All experiments were carried out at 25 °C and pH = 7.

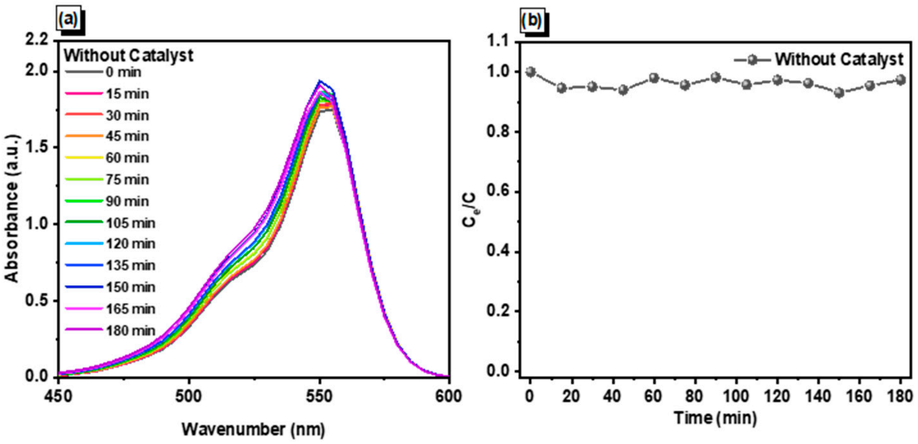

Figure S11. (a) UV-Vis spectra and (b) Photocatalytic efficacy of the control experiment of RhB upon UV and visible light irradiation without catalyst.

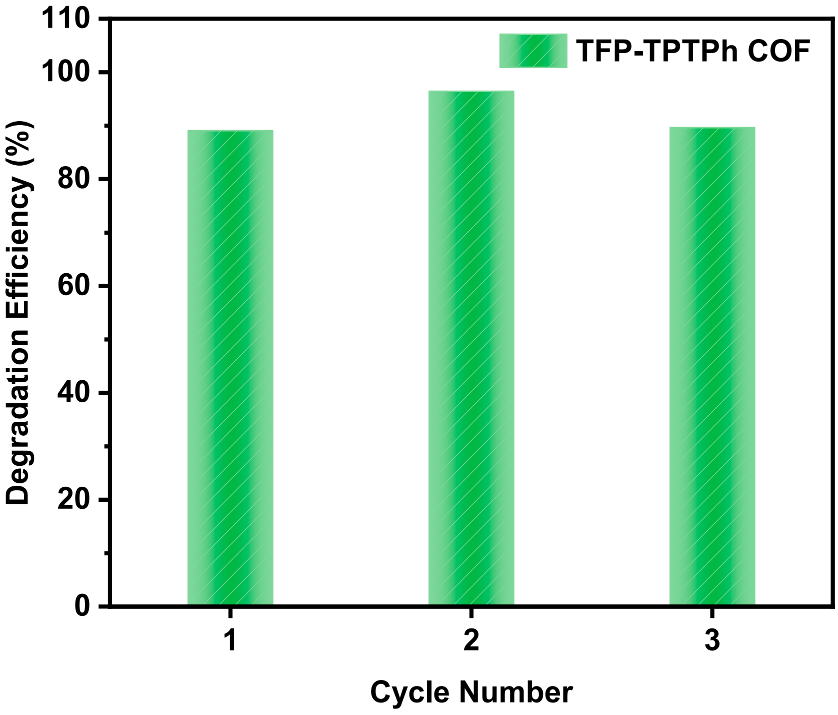

Figure S12. Reusability of TFP-TPTPh COF for the photodegrading of RhB.

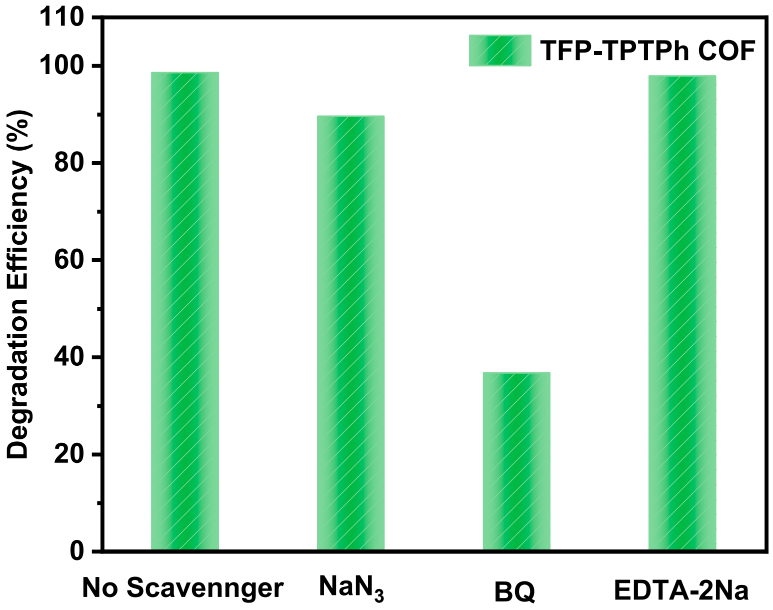

Figure S13. Effect of different scavengers, NaN_3, BQ and EDTA-2Na on the photocatalytic degradation of RhB (10 mg L^{-1}) by TFP-TPTPh COF under UV and visible light irradiation.

Table S5. Photodegradation performance of RhB on the TFP-TPTPh COF, compared with those of other reported materials.

Catalyst	Catalyst amount (mg)	Dye RhB solution	Degradation time (min)	Degradation efficiency (%)	Ref.
COF/g-C_3N_4	20	10 mg L^{-1}, 50 mL	20	~100	[92]
Au@COF	10	10 mg L^{-1}, 100 mL	30	97.3	[93]
POP-1	20	4.8 mg L^{-1}, 10 mL	180	93.3	[94]
Py-POP	10	4.8 mg L^{-1}, 10 mL	80	~100	[95]
TP-COP	100	10 mg L^{-1}, 100 mL	160	95	[96]
COF-JLU19	5	10 mg L^{-1}, 100 mL	6	~100	[97]
Py-BF-CMP	10	75 mg L^{-1}, 50 mL	90	<90.00	[73]
TPE-BF-CMP	10	55 mg L^{-1}, 50 mL	90	~90.00	[73]
TPA-BF-CMP	10	40 mg L^{-1}, 50 mL	90	~90.00	[73]
Py-CMP-1	6	20 mg L^{-1}, 60 mL	90	16.20	[15]
Py-CMP-2	6	20 mg L^{-1}, 60 mL	90	95.77	[15]
TFP-TPT COF	7	10 mg L^{-1}, 56 mL	90	97.02	**This work**

Photocatalytic Hydrogen Evolution Experiments

Photocatalytic H_2 evolution test

The photocatalytic experiments were performed in a 35-mL Pyrex reactor. The reactor was sealed using a rubber septum. In a typical photocatalytic reaction, a COF (2 mg) was dispersed in a mixture of water/DMF (2/1, 10 mL) with 1 M AA as the SED and adjust the pH to 4.0 using 1M KOH. The suspension was purged with Ar for 5 min to remove dissolved air. A 350-W Xe lamp was used as the light source. The light intensity of the Xe lamp was similar to that of the visible light region of standard 1 sun irradiation, as verified using a solar cell. Samples of H_2 were removed with a gas-tight syringe and injected in a Shimadzu GC-2014 gas chromatograph with Ar as the carrier gas. The H_2 was detected using a thermal conductivity detector, with reference to standard H_2 gases of known concentrations.

Quantum efficiency measurements

For the AQY experiments, a catalyst solution was prepared by dispersing a COF (1 mg) in a mixture of water/DMF (2/1, 10 mL) with 1 M AA. The suspension was illuminated

with a 300-W Xe lamp while applying bandpass filters (420, 460, and 500 nm). The formation of H_2 was quantified using a Shimadzu gas chromatograph (GC2014) operated under isothermal conditions, employing a semi capillary column equipped with a thermal conductivity detector. The AQY was calculated as follows:

AQY = [(number of evolved H_2 molecules × 2)/number of incident photons] × 100%:

$$AQY = \frac{N_e}{N_p} \times 100\% = \frac{2 \times M \times N_A}{\frac{E_{total}}{E_{photon}}} \times 100\%$$

$$= \frac{2M \times N_A}{\frac{S \times P \times t}{h \times \frac{c}{\lambda}}} \times 100\% = \frac{2 \times M \times N_A \times h \times c}{S \times P \times t \times \lambda} \times 100\%$$

where M is the amount of H_2 (mol), N_A is Avogadro's constant (6.022×10^{23} mol^{-1}), h is Planck's constant (6.626×10^{-34} J·s), c is the speed of light (3×10^8 m s^{-1}), S is the irradiation area (cm^2), P is the intensity of irradiation light (W cm^{-2}), t is the photoreaction time (s), and λ is the wavelength of the monochromatic light (m).

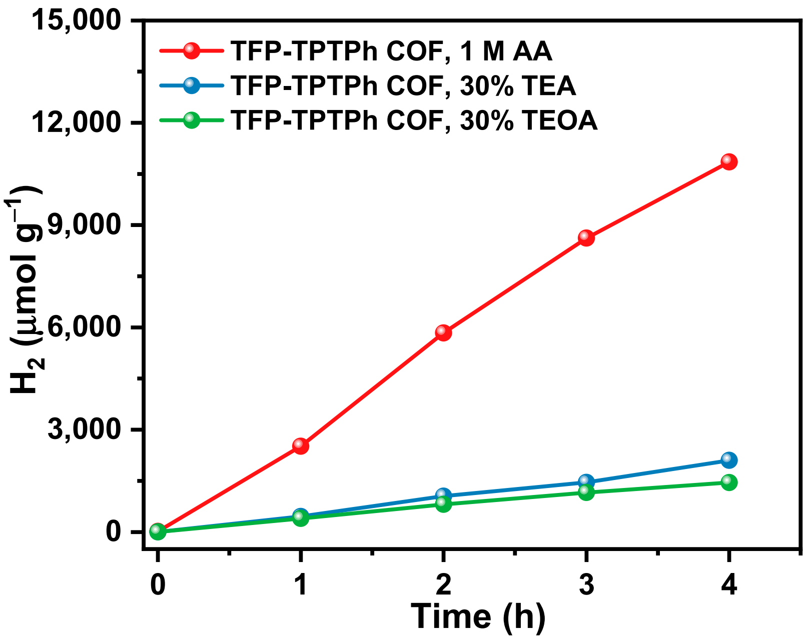

Figure S14. Effect of sacrificial electron donors the hydrogen production.

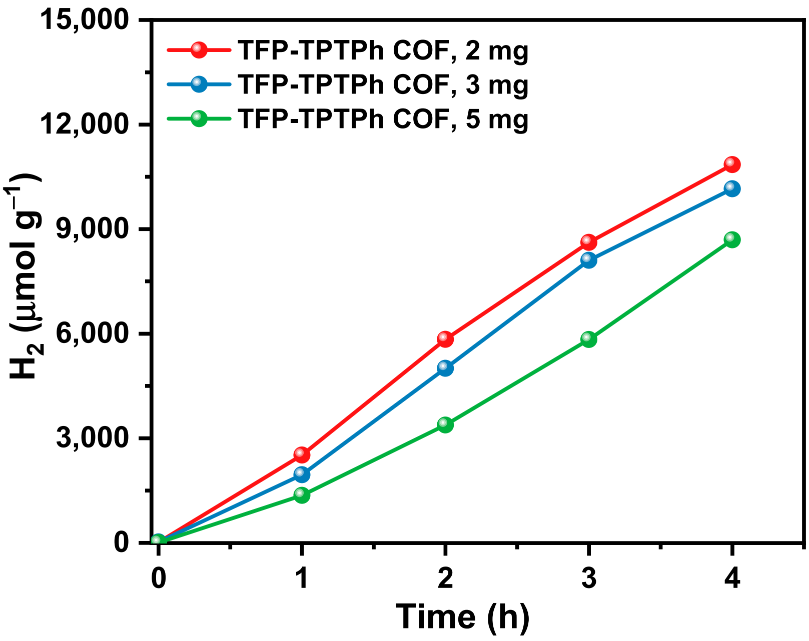

Figure S15. Effect of TFP-TPTPh COF photocatalyst amount on the hydrogen production activity.

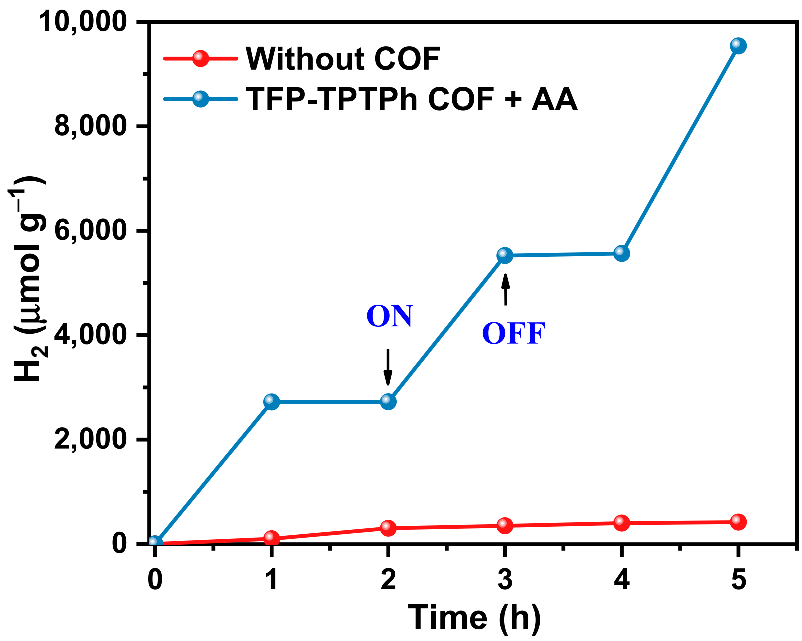

Figure S16. Control experiment of the TFP-TPTPh COF.

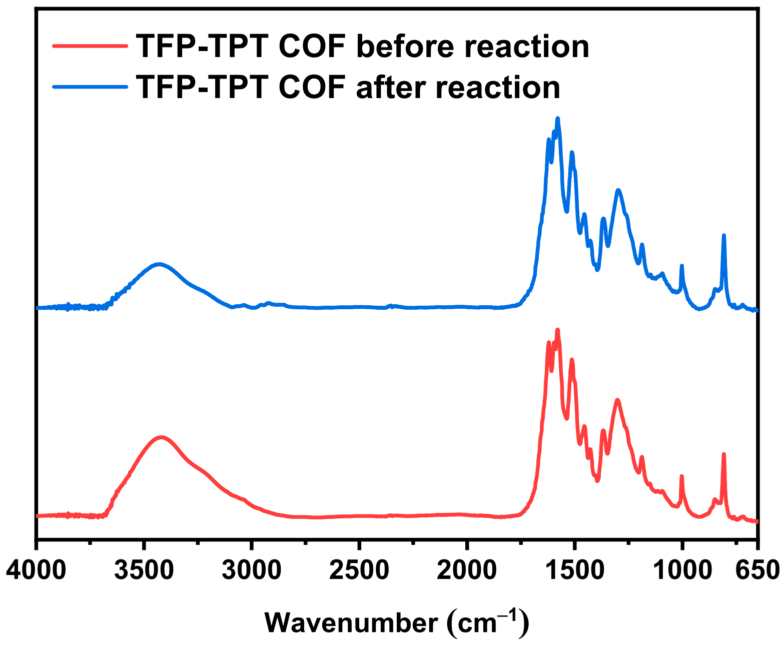

Figure S17. FTIR spectra of TFP-TPTPh COF before and after the photocatalytic H_2 evolution measurement.

Table S6. HERs of TFP -TPTPh COF, compared with those of other reported materials.

Catalyst	SED	HER (μmol g^{-1} h^{-1})	Ref.
BT-COF	0.1 M AA	250	[98]
PyTA-BC-Ph COF	0.1 M AA	420	[99]
BT-TAPTCOF	H_2O/ ascorbic acid, Pt	949	[100]
BtCOF150	H_2O/ TEOA/Pt	750±25	[101]
TP-COF	H_2O/ascorbicacid, Pt	265	[102]

Pt-PVPTPCOF	H_2O/ascorbicacid, Pt	8420	[102]
TpPa COF-NO2	H_2O/ sodium ascorbate, Pt	220	[103]
TpPa COF	H_2O/ sodium ascorbate, Pt	1560	[103]
TpPa COF $(CH_3)_2$	H_2O/ sodium ascorbate, Pt	8330	[103]
TFP-TPTPh	1 M AA	2712	**This work**
